# AP Shadow Net: A Remote Sensing Shadow Removal Network Based on Atmospheric Transport and Poisson’s Equation

**DOI:** 10.3390/e24091301

**Published:** 2022-09-14

**Authors:** Fan Li, Zhiyi Wang, Guoliang He

**Affiliations:** 1 School of Mathematical Sciences, University of Electronic Science and Technology of China, Chengdu 611731, China; 2 UoG-UESTC Joint School, University of Electronic Science and Technology of China, Chengdu 611731, China

**Keywords:** remote sensing image, shadow detection and removal, atmospheric transport model, Poisson equation, deep learning

## Abstract

Shadow is one of the fundamental indicators of remote sensing image which could cause loss or interference of the target data. As a result, the detection and removal of shadow has already been the hotspot of current study because of the complicated background information. In the following passage, a model combining the Atmospheric Transport Model (hereinafter abbreviated as ATM) with the Poisson Equation, AP ShadowNet, is proposed for the shadow detection and removal of remote sensing images by unsupervised learning. This network based on a preprocessing network based on ATM, A Net, and a network based on the Poisson Equation, P Net. Firstly, corresponding mapping between shadow and unshaded area is generated by the ATM. The brightened image will then enter the Confrontation identification in the P Net. Lastly, the reconstructed image is optimized on color consistency and edge transition by Poisson Equation. At present, most shadow removal models based on neural networks are significantly data-driven. Fortunately, by the model in this passage, the unsupervised shadow detection and removal could be released from the data source restrictions from the remote sensing images themselves. By verifying the shadow removal on our model, the result shows a satisfying effect from a both qualitative and quantitative angle. From a qualitative point of view, our results have a prominent effect on tone consistency and removal of detailed shadows. From the quantitative point of view, we adopt the non-reference evaluation indicators: gradient structure similarity (NRSS) and Natural Image Quality Evaluator (NIQE). Combining various evaluation factors such as reasoning speed and memory occupation, it shows that it is outstanding among other current algorithms.

## 1. Introduction

Optical remote sensing images are widely used in environmental monitoring, land surface inversion, ground object classification and various fields. However, the shadow formed by occlusion in the imaging process will cause the loss of original pixel information, which could lead to faulty information. Therefore, it is of great importance to detect and remove the shadows of remote sensing images to guarantee the value of images in research.

At present, a number of researches have been done to remove shadows from simple images by using deep learning networks [1,2,3,4,5,6,7,8,9]. Hieu Le et al. consider that there is a linear relationship between unshaded images and shaded images after estimation and simplification of images. To be more specific, the shaded areas can be brightened by using linear transformation, and then the restored unshaded images can be obtained by fusing the brightened image and the original shadow image [1]. Jin et al. construct a network for hard and soft shadow, combining the traditional entropy physical model with the perceived loss obtained by observing the feature map, to solve the problem of unsupervised shadow removal of a single image [2]. Zhang et al. focused on additional information and used multiple GAN to mine residual and illumination information for shadow removal [3]. However, compared with ordinary images with simple background information, remote sensing images with a complex background have the difficulty of balancing the surface information such as color ratio and high-level semantic features such as the geometric texture of the shadow area. Moreover, remote sensing images cannot obtain corresponding shadow pairing data sets, or even adequate remote sensing images with or without shadow that are suitable for training, which enlightens us to reduce the dependence on data. As a single neural network is difficult to solve the problem, it would be better to combine the traditional physical model and start with the generation principle of the shadow itself.

At the same time, most of the current shadow removal algorithms are problematic in blurring, chromaticity inconsistency and boundary artifact in the restored area. Based on these problems, in this study, a new method of image reconstruction in the shadow area based on the atmospheric transmission model and the generation of countermeasure network are proposed. It also introduces the Poisson Equation to improve the spatial smoothness and continuous texture similarity at the boundary of the shadow area to optimize the local area. As a result, it can detect and remove shadows of remote sensing images in detail without interfere the original texture and background features of the shadow area.

Because of the training of deep learning need work based on the physical principle, our method has certain robustness and wide applicability applied to most aerial remote sensing images, and the range can include urban buildings, road traffic, natural vegetation and other fields. Compared with other methods, it performs better on the maintenance of the color while brighten the shadow area. It shows better effects on deeper colors and more subtle shadows and shows more advanced performance in qualitative and quantitative evaluation experiments. In addition, this research can be further extended to other fields, which is of great significance for military reconnaissance and detection based on remote sensing images, urban construction for traffic planning and other various applications. The specific contributions of this method can be summarized as follows:

- Propose an unsupervised shadow removal method for remote sensing images to overcome the difficulties of obtaining adequate shadow data sets of remote sensing images;
- Apply the method of Atmospheric Transmission Model combined with neural network based on the physical model, enhancing the robustness of neural network training, releasing the strict restrictions of traditional methods on natural conditions such as satellites and improving the versatility under different conditions;
- Take the Preliminary Restoration map obtained by the atmospheric transmission model as the ground truth, addressing the problems of slow convergence and insufficient mapping constraints caused by the traditional generation of “unpaired” images of the countermeasure network, and improving the efficiency of shadow removal;
- Use chromaticity consistency loss, structure perception loss and Poisson guidance loss based on Poisson equation, optimizing effects on detailed shadows, maintaining color consistency and reducing boundary effects.

## 2. Related Work

In order to mitigate the interference of shadow on remote sensing images and improve the efficiency of utilization of images, researchers are trying to improve shadow processing technology of remote sensing images from various angles [10,11,12,13,14,15]. Our work is a new attempt to reform the shadow images by combining the Atmospheric Physical Transmission model with the generation of confrontation network. The following passage will review the work related to shadow removal, including traditional methods and neural network methods.

Traditional methods could be classified into image enhancement based and shadow compensation based on model. Image enhancement processing is to consider shadow as a special noise to analyze the characteristics of the shadow area in terms of spectrum and texture and then using image enhancement or denoising to compensate. Wang et al. [16] proposed a method based on Luminance based Multi-Scale Retinex (LMSR) [17], which first compensates the brightness component, then synthesizes it with the primary color component and then converts it to RGB space. Tian et al. combined gray-scale linear mapping transformation and histogram matching method to compensate. Li [18] and Zhou [19] proposed a gray-scale mapping of shadow homogenous region [20,21], by constructing the mapping between shadow pixels and shadow homogenous region pixels through improved gamma transformation repair the shadow region.

Ye et al. [22] used Minkowski normal form to estimate the light source color and non-shadow areas of the image constructing a mapping to simulate the lighting conditions of non-shadow for compensation. From the theoretical angle of radiation transmission, Guo et al. considered the transmission of solar energy and proposed a shadow removal theory based on the energy information compensation model.

However, most methods based solely on physical models could work well with adequate information of satellite, surface and atmospheric parameters, but their performance are highly limited by the absence of prior conditions such as satellite information. To resolve these barriers, methods based on deep learning, such as pixel fusion using fusion network, u-net, flow net [23], the method of generating confrontation network [2,8,24,25,26], and the method of improving compensation based on CNN basic network [27] are proposed for shadow area image reconstruction. As for the compensation of the shadow area, the essence is allied to the enhancement of the low illumination image. Combined with the image enhancement processing idea mentioned above, the shadow removal task is similar to the removal of “noise” under conditions like thick clouds and thick fog. Li et al. [28] proposed a dark light image enhancement method based on zero reference depth curve estimation, which converts the enhancement task into the estimation of specific function, and measures the subtle enhancement quality by designing a group of differentiable non reference losses to drive the learning; the IDE proposed by Ju [29] and the IDGCP method proposed by and [30] et al. improved on the IDE are based on the atmospheric scattering model, extracting the depth ratio from the original haze image and the uniform virtual transformation. Finally, the global strategy and visual indicators to restore the scene reflectivity are used to restore the haze image.

Single traditional models or neural network methods have limitations on the shadow removal task of remote sensing images. Inspired by the advanced methods above, we combine traditional physical models, neural network, denoising and image restoration to propose an unsupervised remote sensing image shadow removal network: AP Net, which combines an atmospheric transmission model with the Poisson equation. A Net uses the atmospheric physical transport model to estimate the corresponding mapping parameters between shadow and non-shadow maps. The original shadow image and the preliminary restored image are transferred to P Net, while the images with different shadow conditions under similar background are used for confrontation reconstruction. It can address the obstacles of slow convergence and insufficient mapping constraints caused by the “unpaired” images of the original network and improve the efficiency of shadow removal.

To solve the difficulty that the inconsistency between color intensity in the shadow repair area of the reconstructed image and that of the surrounding area with artifacts, we introduce chromaticity consistency loss in P Net. Additionally, because of the similarity of the color gradient and shadow boundary in the restored area of the reconstructed image and that in the surrounding area, the continuity of the pixel characteristics at the boundary between the shadow and the non-shadow area, we adopt the Poisson partial differential equation to solve the given Dirichlet boundary condition, and use the color gradient to replace the color intensity to produce a more reasonable effect. Beside the model above, MS-SSIM L1 loss is used to retain high-frequency information such as texture details in the original shadow area while maintaining color brightness characteristics.

**Figure 1:**
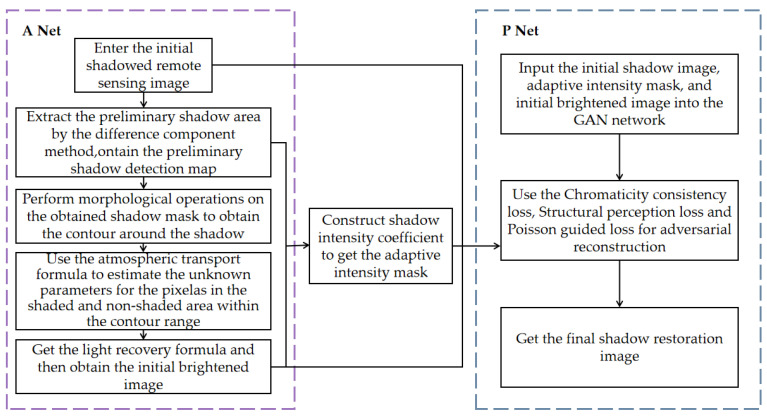
The flow chart of remote sensing image shadow detection and removal model based on AP Net.

The flow chart of the whole shadow detection and removal modeling method is shown in Figure 1 below:

## 3. Proposed Method

The traditional physical shadow removal model has difficulties in obtaining parameters and processing a large number of images as a whole due to the large amount of calculation. However, another kind of method based on deep network lacks the interpretability of the internal mechanism. Therefore, we propose a method combining traditional physical models and deep networks, using a simplified atmospheric transport model to construct the relationship between non-shadow maps and shadow maps. For the case that the atmospheric attenuation coefficient and atmospheric transmittance coefficient are more complicated and difficult to obtain in the original function, A Net is used to learn the corresponding parameters. After the parameters are determined, the preliminary shadow restoration and brightening map can be calculated according to the physical model formula.

In order to solve the obstacles that remote sensing shadow images lack ground truth, the above-obtained incremental images are passed as reference images instead of ground truth to P Net, then used in the Cycle Gan for further learning, and finally a restored image that satisfies the conditions of the discriminator could be obtained from the network.

### 3.1. A Net: Simplifying the Atmospheric Transport Model to Construct Unshaded Maps

#### 3.1.1. Difference Component Method to Extract the Preliminary Shadow Area

According to the physical principle, Shadows is generated by higher objects blocking direct sunlight, so that the characteristics of dark targets corresponding to specific shadows are determined by the amount of sunlight radiated to the target and reflected from the target objects. According to the research of GUO et al. [31], the shadows of remote sensing images are mainly concentrated in the interval from the visible light to near-infrared bands where the proportion of ambient reflected light is very small [31]. Shadowed areas are missing most of the direct light, which led to higher saturation and lower lightness in the HSI color space compared to other objects such as roads, vegetation, and buildings [32]. Based on this feature, we construct a normalized saturation difference index (SI) and use the maximum inter-class variance algorithm to segment and extract shadows from the SI matrix calculated from the image. For each image pixel, SI is calculated by the formula as follows:

(1) $$SI=\frac{S-I}{S+I}$$

where *S* and *I* are the corresponding components of the *I* space respectively.

#### 3.1.2. Shadow Area Recovery of Remote Sensing Images

It is believed that a remote sensing shadow image is a shadowless remote sensing image combined with atmospheric effects such as direct light and reflected light. To be more specific, there is a mapping relationship between shadow images and non-shadow images:

(2) $$\psi :\;I_{sf}(x,y)\to I_{s}(x,y)$$

where $I_{sf}$ is the original unshaded remote sensing image, $I_{s}$ is the shaded remote sensing image, and ψ is a combination of direct, reflected, and scattered atmospheric effects.

Remote sensing images are mainly formed by satellite sensors receiving radiation or reflection information from the ground. Since large objects block most of the direct sunlight, the information received from direct incident light in shadowed areas is basically negligible. As a result, the reflection and scattering of atmospheric space becomes the main factors. Except for the part of the solar radiation that is directly scattered by the atmosphere into space, the contribution of the ground object to the information received by the sensor can be roughly divided into [31]:

- Reached the ground object and scattered by the atmosphere, then directly reflected into the sensor:
  (3) $$t\left(\mu_{S}\right)\rho e^{-\tau /\mu_{V}}$$
- Reached the object and catered by the atmosphere, then scattered by the atmosphere to the sensor as well as the part where the ground and the atmosphere are scattered with each other for multiple times to reach the sensor:
  (4) $$t\left(\mu_{V}\right)\tilde{\rho }t\left(\mu_{S}\right)+\frac{\left(e^{-\tau /\mu_{S}}+t\left(\mu_{S}\right)\right)\left(e^{-\tau /\mu_{V}}+t\left(\mu_{V}\right)\right)S{\tilde{\rho }}^{2}}{1-S\tilde{\rho }}$$
  where $\mu_{V}$ is the cosine of the solar zenith angle, $\mu_{S}$ is the cosine of the zenith angle of the remote sensor, ρ and $\tilde{\rho }$ are the terrestrial hemispheric reflectance of the corresponding processes, τ is the atmospheric attenuation coefficient, S is the top-level atmospheric reflectance, *t*($\mu_{S}$) is the atmospheric transmittance of light reaching the object, and *t*($\mu_{V}$) is the atmospheric transmittance of light directed or reflected from the object to the sensor.

The inverse mapping of atmospheric transport is represented by the ratio of the amount of radiation received in non-shadowed to the shadowed condition, which can be written as [31]:

(5) $$H(\mu_{V},\mu_{S})=e^{-\tau /\mu_{V}}\rho_{S}e^{-\tau /\mu_{S}}+e^{-\tau /\mu_{V}}t\left(\mu_{S}\right)\rho +e^{-\tau /\mu_{V}}\rho^{\prime }t\left(\mu_{V}\right)+t\left(\mu_{S}\right)t\left(\mu_{V}\right)\tilde{\rho }\;$$

(6) $$K(\mu_{V},\mu_{S})=\frac{\left(e^{-\tau /\mu_{S}}+t\left(\mu_{S}\right)\right)\left(e^{-\tau /\mu_{V}}+t\left(\mu_{V}\right)\right)S{\tilde{\rho }}^{2}}{1-S\tilde{\rho }}$$

(7) $$G(\mu_{V},\mu_{S})=t\left(\mu_{S}\right)\rho e^{-\tau /\mu_{V}}t\left(\mu_{V}\right)t\left(\mu_{S}\right)\tilde{\rho }+\frac{\left(e^{-\tau /\mu_{S}}+t\left(\mu_{S}\right)\right)\left(e^{-\tau /\mu_{V}}+t\left(\mu_{V}\right)\right)S{\tilde{\rho }}^{2}}{1-S\tilde{\rho }}$$

so, we have

(8) $$\left|\psi^{-1}\right|=\frac{H(\mu_{V},\mu_{S})+K(\mu_{V},\mu_{S})}{G(\mu_{V},\mu_{S})}$$

where $\rho_{s}$ is the ground reflectance and ρ′ is the ground hemispheric reflectance scattered to the atmosphere by the ground.

The visible wavelength scattered by the ground and the atmosphere for multiple times has been attenuated and compared with the direct sunlight and atmospheric scattered light, it is less significant, so the $\frac{\left(e^{-\tau /\mu_{S}}+t\left(\mu_{S}\right)\right)\left(e^{-\tau /\mu_{V}}+t\left(\mu_{V}\right)\right)S{\tilde{\rho }}^{2}}{1-S\tilde{\rho }}$ part will do reasonable neglect.

The reflection of electromagnetic waves includes specular reflection, diffuse reflection, which reflects incident light in all directions from entire surface evenly, and directional reflection, which reflects in all directions but not uniform. Since most remote sensing images contain buildings or vegetation, and the wavelength of such natural surfaces is rough enough, the reflected radiance can be approximately regarded as not changing with the observation angle, which can be assumed that the observed ground the object target is a homogeneous Lambertian body, so it follows: $\tilde{\rho }=\rho$.

Therefore, referring to the existing remote sensing shadow images, a pure shadow-free image can be obtained only by solving the inverse mapping of the atmosphere comprehensive action. Since the deep network needs to be used for subsequent learning, its certain robustness will make a sense on improving the removal effect. Therefore, in order to solve the complicity of the calculation, the model for solving the inverse mapping of atmospheric transport is simplified as follows:

(9) $$\left|\psi^{-1}\right|=\frac{t\left(\mu_{S}\right)+e^{-\tau /\mu_{S}}}{t\left(\mu_{S}\right)}$$

According to the experiments, the effect of radiation on the removal of image shadows is not significant relative to the transmission mapping, so the initial operation only retains the transmission mapping function, written as:

(10) $$I_{sf}(x,y)=I_{s}(x,y)\cdot \psi^{-1}(x,y)$$

For the unknown parameter $\mu_{S}$ and τ above, several expansion operations and then subtracted are performed to the original image to obtain a contour map for preliminary detection of shadows. The least squares method is used to compare with the area around the shadow to learn the parameters. Formula (10) is brought in to restore the initial brightened image after calculation.

### 3.2. Adaptive Intensity Shadow Masking

The convolution could be simplified to the product form, and the atmospheric transport inverse mapping simplified model can be used to perform a preliminary operation. The initial brightened image is noted as: $I_{bri}$.

Because of the complexity of the background information and the pixel information loss brought by the shadow in the occluded area, we propose a shadow mask construction method in order to reduce the loss of sharpness and enhance removing effect of penumbra in the restoration area. The method normalizes the original shadow area pixel by pixel to describe the shadow intensity of each point and retains the details of the background on the basis of the original boundary.

The shadow intensity coefficient is constructed as the difference between the original shadow image and the brightened image as follows:

(11) $$I_{mat\;}=255-\left[\alpha \cdot \left(255-I_{mask\;}\right)+I_{bri\;}\right]$$

where $I_{mat}$ is the adaptive intensity mask and $I_{mask}$ is the original binary shadow mask.

### 3.3. P Net: Adversarial Reconstruction to Obtain Final Shadow-Free Map

#### 3.3.1. Overall Framework

The overall network can be divided into two parts. One part is A Net for obtaining mapping parameters to construct brightened images. Specifically, we use the least squares method to estimate the parameters. The other part is P Net for finally generating shadow-free images. The input of the A Net part is the original shadow image and the initial mask. The morphological algorithm performs several expansion operations on the boundary area of the initial mask. Then the contour area around the shadow can be obtained by subtraction.

**Figure 2:**
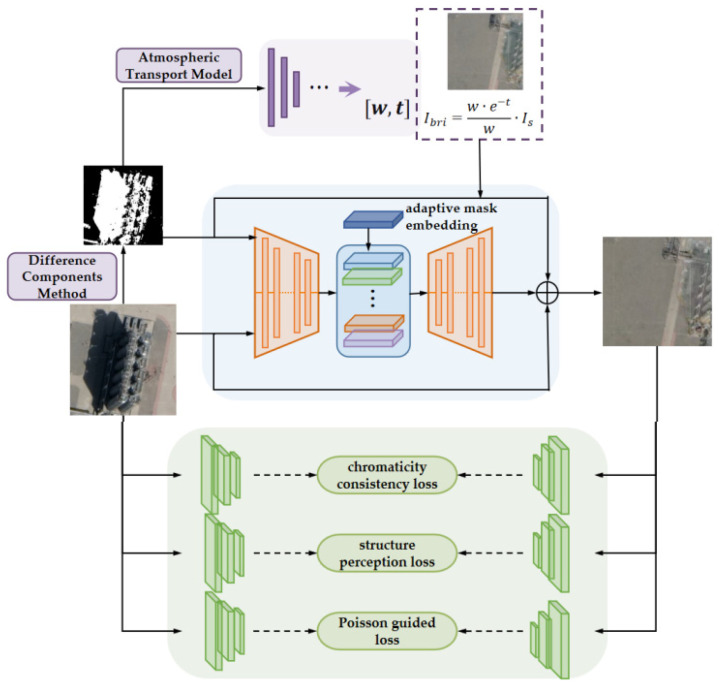
The diagram of AP Net network structure.

The initial brightening image can be calculated by the mapping parameters from A Net and the Formula (10). The process image with the adaptive strength mask was modified in P Net on the basis of the STC Gan model [15]. With Cycle Gan as the prototype, the generator part integrates the chromaticity consistency loss, the structure perception loss, and the embedding layer is added to consider the shadow intensity when generating the adaptive mask. A Poisson equation guided loss is added to judge the generated images whether hold more natural shadow boundaries the discriminator stage. The structure of AP Net network is shown in Figure 2 below:

#### 3.3.2. Loss Function

In the network structure of the loss functions, in addition to the adversary losses contained in the original network, consistent losses and identity losses, chromaticity consistency loss, structure perception loss and Poisson guided loss are also introduced in the network.

- *Chromaticity consistency loss*

The main difficulties of many studies on shadow removal are the inconsistency of regional chromaticity. There are methods of constructing compensation coefficients [33], bilinear difference with surrounding areas [34], and color uniformity based on Wallis filtering [35]. In this paper, a chrominance loss function is proposed by constraining the pixel values of the three channels of *R*, *G*, and *B* in the generated image, which try to be close to the brightened image after illumination compensation to achieve the effect of maintaining color consistency. Specifically, it can be expressed as:

(12) $$L_{chroma\;}=\sqrt{\sum_{c}{\left\|Z_{sf_{c}}-Z_{bri_{c}}\right\|}^{2}}$$

where c ϵ {R,G,B} represents the corresponding color channel of the image, $Z_{sf_{c}}$ and $Z_{bri_{c}}$ are the pixel values of the color channel of the generated image and the brightened image, respectively.

- *Structural perception loss*

For the reconstruction of the shadow area, the desired goal is to improve the brightness of the shadow area and enhance its detailed texture, while the distortion degree of the reconstructed image can be measured by the structural similarity index *SSIM*. In addition, the perception-based model is more in line with the human eye’s ability to eliminate intuitive need for shadows. *SSIM* mainly considers the features of the image in terms of brightness, contrast and structure. The brightness is measured by the average gray value of the image, the contrast is characterized by an unbiased estimate of the gray standard deviation, and the structure is measured by the normalized correlation coefficient. For a certain pixel P, the specific formula for calculating *SSIM* can be written as:

(13) $$SSIM(P)=\frac{2\mu_{x}\mu_{y}+C_{1}}{\mu_{x}^{2}+\mu_{y}^{2}+C_{1}}\cdot \frac{2\sigma_{xy}+C_{2}}{\sigma_{x}^{2}+\sigma_{y}^{2}+C_{2}}$$

denote the validation image and the reference image by *X* and *Y*, respectively, $\mu_{*}$ is the mean value of the corresponding *X* or *Y*, $\sigma_{*}$ is the standard deviation, and $\sigma_{xy}$ is the covariance of *X* and *Y*.

The mean and variance of the image are calculated by Gaussian kernel and image convolution, where *P* is defined as the set of patch pixels and *N* as the number of pixels. The *SSIM* loss can be written as:

(14) $$L^{SSIM}(P)=\frac{1}{\;N}\sum_{p\in P}1-SSIM(p)$$

*SSIM* loss can maintain better high-frequency detail but is insensitive to smooth variations that can cause color shifts or brightness changes, while *L*_1_ Loss can preserve region color and brightness but ignores local structure. Thus, assume *SSIM-L*_1_ Loss is a combination of the two constructs, which is expressed as follows:

(15) $$L_{SSIM-L1}=\gamma \cdot L^{SSIM}+(1-\gamma )\cdot L_{1}$$

where

(16) $$L_{1}(P)=\frac{1}{\;N}\sum_{p\in P}|x(p)-y(p)|$$

To balance the two loss values, the γ parameter in Equation (15) was empirically set to 0.84 [36].

- *Poisson guided loss*

A common problem faced by the existing shadow removal methods is that the transition at the border of the shadow area is unnatural and with artifacts. Taking the method in this paper as an example, we combine the non-shadow area of the original image with the shadow area of the incremental image. The idea of reconstructing shadow-free images is essentially similar to image fusion.

It is of great significance to generate a smooth and natural shadow boundary, that is, to keep the texture and color consistency of the fusion boundary. Rez P et al. found that when the images are fused, the color intensity in the masked area of the generated image is inconsistent with the source image, but the color gradients of the two are basically the same. Therefore, we replace the color intensity with the color gradient, which can produce a more realistic effect [37]. The essence of our goal is also similar to solving Poisson partial differential equations that given Dirichlet boundary conditions.

We define the closed subset *S* as the image definition domain, also the original remote sensing shadow image. Ω is the closed subset of *S*, which represents the shadow area to be reconstructed. In addition, $f^{*}$ is the known scalar function defined on the boundary and outside of domain Ω, representing the known information of the non-shadow area. Taking the brightened image as the guide map, g is the known scalar function of the region to be fused. f is the unknown scalar function defined inside the domain Ω. To reconstruct the information of the shadow area and keep the gradient texture information as well as satisfy the natural transition, it is necessary to solve the unknown scalar function f, and at the same time satisfy the condition that hold f and $f^{*}$ the same gradient on the boundary of the domain Ω. The formula can be written as:

(17) $$L_{Possion\;}=\iint_{\Omega }|\nabla f-\nabla g{|}^{2}$$

with

(18) $$\{\begin{array}{c} {f|}_{\partial \Omega }={f^{*}|}_{\partial \Omega }\\ \Delta f=\Delta g=\mathrm{div}(\nabla g) \end{array}$$

- *Overall loss*

Based on the model above, the overall loss function is as followed:

(19) $$L_{total\;}=L_{chroma\;}+L_{Mix\;}+L_{Possion\;}$$

## 4. Experiment

**Figure 3:**
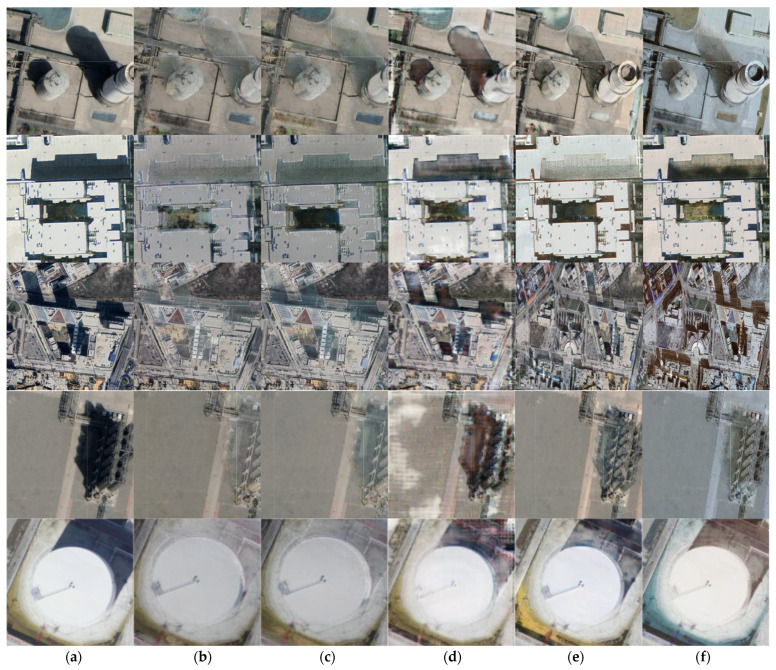
The comparison effect of the method in this paper and other methods in removing shadows from remote sensing images, where (**a**) is the original input image, (**b**) is the effect of our method, (**c**) is the ST-CGan model effect, and (**d**) is the Mask Shadow Gan model renderings, (**e**) is DC Shadow Net model renderings, (**f**) is Ghost Free Net model renderings.

To evaluate our method, we trained on 3747 images and finally tested on 2702 images using our dataset. The experimental results are shown in Figure 3 below:

It can be seen from the experimental results that our method has a better removal effect on images having darker shadows and the shadows with fine and large numbers caused by complex objects. Compared with ST-CGan model and Ghost Free Net with a problem of tone change in the restored shadow area or the overall image, our method approximates the values of the R, G, B three channels from the pixel level, providing a better restoration effect of the shadow area which is closer to the surrounding non-shadow area and conforms to the natural scene. For the Mask Shadow Gan model and DC Shadow Net, the use of the Dirichlet boundary condition does a good job on the removal of dark shadows and the transition of borders. At the same time, it is worth noting that compared with other methods, our model can detect some smaller shadow areas and make corresponding restorations.

Due to the lack of ground truth in remote sensing shadow images, evaluation indicators such as MSE and PSNR that require reference images are not suitable to be used. In this paper, we use the Gradient Structure Similarity (NRSS) and Natural Image Quality Evaluator (NIQE) to measure the quality of shadow removal images.

- *Gradient Structure Similarity (NRSS)*

A major factor affecting the quality of the restoration structure of remote sensing images is the details in the shadow, the clarity of image restoration can be measured according to the amount of high-frequency information contained in the target shadow area after processing. In the current methods, Liang et al. [38] used the standard deviation of the gradient profile to represent the sharpness of a point, established the sharpness histogram of all edge points, and calculated the image blurriness accordingly. Saad et al. [39] proposed a Blind image integrity notator using DCT Statistics (BLIINDS) to predict image quality based on DCT transform, anisotropic entropy and multivariate Gaussian probability model. In this paper, the clarity of details of shadow removal is measured by the gradient structure similarity *NRSS* index [40]. Firstly, low-pass filtering is performed on the original image to obtain the reference image, and then the structural similarity in the target area between the reference image and the original image to be evaluated is calculated. The indicators are defined as:

(20) $$NRSS=1-\frac{1}{N}\sum_{i=1}^{N}SSIM(p)$$

where *N* is the numbers of image blocks with the most abundant gradient information selected in the gradient image G.

- *Natural Image Quality Evaluator (NIQE)*

In the image inpainting task, some evaluation indicators such as PSNR can be closer to the original image at the pixel level, but images with high similar indicators may not conform to the visual habits of the human eye, which cannot prove that the effect of inpainting and reconstruction is more effective. Since human eye is more sensitive to areas with higher contrast ratios in the image, we use the Natural Image Quality Evaluator index [41] (NIQE), fitting it into the Multivariate Gaussian (MVG) model based on the “quality perception” features and regard the corresponding distance as the quality of reconstruction.

The NIQE algorithm uses the maximum likelihood method to estimate the mean and variance matrix of the MVG model, extracts the spatial domain features of the restored image, and calculates the distance between the mean and variance parameters of the restored image and the natural image MVG fitting as the evaluation index:

(21) $$D\left(v_{1},v_{2},\sum_{1},\sum_{2}\right)=\sqrt{{\left(v_{1}-v_{2}\right)}^{T}{\left(\frac{\sum_{1}+\sum_{2}}{2}\right)}^{-1}\left(v_{1}-v_{2}\right)}$$

among them, $v_{1},v_{2},{\sum^{\;}}_{1},{\sum^{\;}}_{2}$, respectively, represent the mean and variance matrix of the MVG model obtained by fitting the natural image and the distorted image.

We use gradient structure similarity (*NRSS*) to assess the realism of the generated shadow-free images and Natural Image Quality Evaluator (NIQE) to measure the perceptual similarity, where the lower values show the better performance. The specific results of comparison with several advanced shadow removal methods are shown in Table 1 below:

The experimental result shows that our model performs better than other competitors regardless of whether it is evaluated at the pixel level or perceptual level. In comparison, the gradient structure similarity is improved by at least 5.67%, and the Natural Image Quality Evaluator is improved by at least 3.62%, which proves the effectiveness of our model.

## 5. Ablation Studies

**Figure 4:**
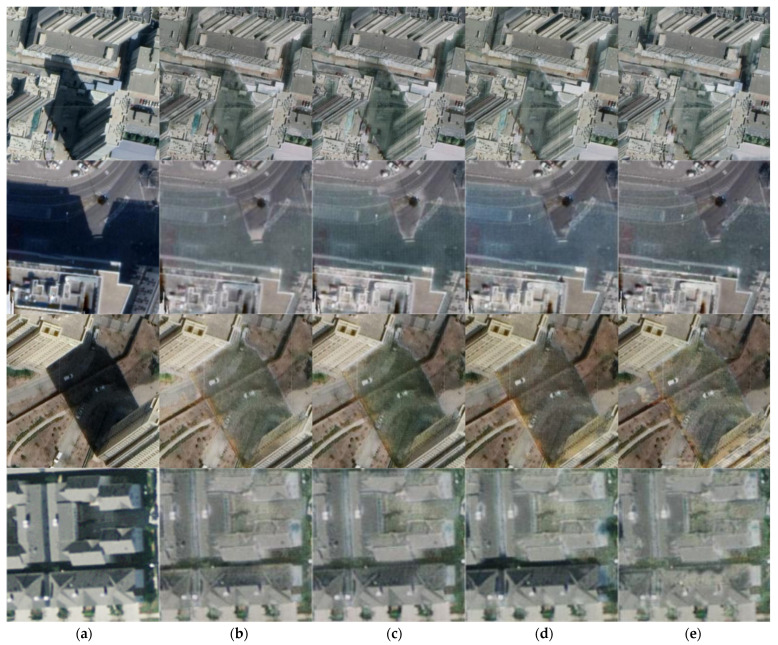
The result of the ablation experiment after removing some components, where (**a**) is the original input image, (**b**) is the result of removing the chromatic consistency loss. (**c**) is the effect of removing the structural perception loss, (**d**) is the result of removing the Poisson guided loss, and (**e**) is the shadow-free picture that the complete model generates.

We conducted an ablation study to analyze the effectiveness of various components of our method, such as: chromaticity consistency loss, structure perception loss and Poisson guided loss. The corresponding results are shown in Figure 4 below:

The specific quantitative comparison results are in Table 2 below:

According to the experimental results, at the gradient similarity level, the removal of the structural perception loss has the greatest impact on the removal effect, while at the perception level, the difference between the results after removing the chromaticity consistency loss is significant. Additionally, the shaded area and the non-shaded area have basically the same impact on the NRSS indicator. However, in the calculation process of NIQE, the shadow area fluctuates by 0.7162, and the non-shadow area fluctuates by 0.5519, indicating that the transformation of the model to the shadow area of the image is the main factor causing the improvement of perception.

## 6. Conclusions

We propose AP Net, a network for removing shadows of remote sensing images, which combines atmospheric physical transport models with chromatic consistency, structural features, and Poisson equation-based losses. Unsupervised training could be suitable for processing remote sensing images with complex backgrounds. In addition, supported by physical principles, the applicability of the model is expanded to address the limitations of the dataset. Combining traditional physical models and GAN network, the method in this paper achieves more perfect results in the color consistency, edge smoothing and fine shadow detection and compensation of shadow areas. Different degrees of improvement have also been gained in Gradient Structural Similarity (NRSS) and Natural Image Quality Evaluation Index (NIQE). Moreover, we confirm the validity of each component of the network by comparing experiments with other shadow removal methods on the unified dataset, the results show that the removal effect of our model is better than other advanced models.

## Figures and Tables

**Table 1:** Quantitative comparison of shadow removal results for the dataset.

Method	Area	NRSS	NIQE
Ours	Shadow	0.9621	15.7264
	Non-shadow	0.9642	16.5147
	All	0.9343	4.5851
ST-CGan [12]	Shadow	0.9633	16.2860
	Non-shadow	0.9693	17.6712
	All	0.9433	4.6267
Mask Shadow Gan [3]	Shadow	0.9806	28.5165
	Non-shadow	0.9815	37.3405
	All	0.9463	4.6211
DC-Shadow Net [2]	Shadow	0.9651	17.1019
	Non-shadow	0.9752	17.9761
	All	0.9399	5.1012
Ghost Free Net [9]	Shadow	0.9681	36.6969
	Non-shadow	0.9846	40.6417
	All	0.9482	4.8993

**Table 2:** Quantitative comparison of comparative results of ablation studies with different components.

Method	Area	NRSS	NIQE
Ours	Shadow	0.9621	15.7264
	Non-shadow	0.9642	16.5147
	All	0.9363	4.5851
$w/o\;L_{chroma}$	Shadow	0.9673	16.4426
	Non-shadow	0.9698	17.0666
	All	0.9408	4.4880
$w/o\;L_{Mix}$	Shadow	0.9690	16.9150
	Non-shadow	0.9712	17.4450
	All	0.9420	4.6016
$w/o\;L_{Possion}$	Shadow	0.9651	15.6853
	Non-shadow	0.9696	16.4976
	All	0.9388	4.5631

## Data Availability

Not applicable.
